# Venovenous Extracorporeal Membrane Oxygenation for Acute Respiratory Distress Syndrome Due to Severe Fever With Thrombocytopenia Syndrome

**DOI:** 10.7759/cureus.39138

**Published:** 2023-05-17

**Authors:** Nobu Fukumoto, Kyohei Miyamoto, Tsuyoshi Nakashima, Seiya Kato

**Affiliations:** 1 Department of Emergency and Critical Care Medicine, Wakayama Medical University, Wakayama, JPN

**Keywords:** sfts, venovenous extracorporeal membrane oxygenation, sepsis, multiple organ failure, acute respiratory distress syndrome

## Abstract

Few cases of acute respiratory distress syndrome (ARDS) in severe fever with thrombocytopenia syndrome (SFTS) have been treated with venovenous extracorporeal membrane oxygenation (VV-ECMO), and its role remains unclear. A 73-year-old Japanese woman presented with multiple organ failure (MOF) due to SFTS, including liver, neural, hematologic, renal, and ARDS. VV-ECMO for refractory hypoxemia under lung-protective ventilation with prone positioning led to gradual respiratory improvement, and she was successfully weaned on the 19th day of hospitalization. However, she died from persistent MOF on the 60th day of hospitalization. VV-ECMO contributed to recovery from ARDS but not from the ultimate cause of death, i.e., MOF. SFTS could have variable MOFs with different disease trajectories, which influence the decision for VV-ECMO.

## Introduction

Severe fever with thrombocytopenia syndrome (SFTS) is an emerging infection endemic to East Asia, caused by the SFTS virus. Previous studies have reported a mortality rate of approximately 30% [1]. The cause of death is reported to be rapidly progressing multiple organ failure (MOF) due to a severe cytokine storm, accompanied by a marked elevation of liver enzymes or lactate dehydrogenase [2]. The incidence of respiratory failure seems to be relatively low; however, acute respiratory distress syndrome (ARDS) has been reported to be associated with a higher mortality rate [3].

Venovenous extracorporeal membrane oxygenation (VV-ECMO) is a life-saving measure for refractory ARDS. Recent studies have suggested that patients with severe ARDS caused by viral infections, including influenza and coronavirus disease 2019 (COVID-19), could be good candidates for VV-ECMO [4,5]. Few reports of this exist in SFTS. Here, we present a case of SFTS complicated by severe ARDS that was treated with VV-ECMO.

## Case presentation

A previously healthy 73-year-old woman was admitted to our hospital with a fever and MOF. She was initially admitted elsewhere with a five-day history of anorexia, malaise, fever, and diarrhea and was referred to our tertiary care hospital for MOF as she developed altered mental status and hypotension. Her history revealed that she mowed weeds in forests and mountains.

On arrival, her blood pressure was 80/50 mmHg, heart rate was 91 bpm, Glasgow Coma Scale (GCS) score was E3V4M6, body temperature was 37.2°C, respiratory rate was 23 breaths per minute, and oxygen saturation was 97% on room air. Examination revealed epistaxis and oral bleeding but no skin rash or traces of tick bites. Laboratory testing revealed pancytopenia, elevated liver enzymes and lactate dehydrogenase, coagulopathy, and renal dysfunction related to underlying disseminated intravascular coagulation (Table 1). Radiological examination, including brain, chest, and abdominal computed tomography, revealed no significant findings. Pathological examination of bone marrow aspirate revealed mild hemophagocytosis.

**Table 1:** Patient’s laboratory findings on admission * Blood lactate levels were measured using arterial blood gas analysis.

Variable	Value	Reference value
White cell count (per mm^3^)	2,920	3,300–9,000
Neutrophil count (per mm^3^)	1,250	1,500–7,500
Lymphocyte count (per mm^3^)	1,460	1,000–4,000
Hemoglobin (g/dl)	10.3	11.6–14.8
Hematocrit (%)	30.5	35.1–44.4
Platelet count (per mm^3^)	15,000	158,000–348,000
Sodium (mmol/l)	135	138–145
Potassium (mmol/l)	3.8	3.6–4.8
Chloride (mmol/l)	108	101–108
Creatine kinase (IU/L)	309	41–153
Urea nitrogen (mg/dl)	18.2	8–20
Creatinine (mg/dl)	1.14	0.46–0.79
Aspartate aminotransferase (IU/l)	624	13–30
Alanine aminotransferase (IU/l)	221	7–23
Lactate dehydrogenase (IU/l)	1,954	115–229
Total bilirubin (mg/dl)	1.4	0.3–1.2
International normalized ratio	1.15	0.8–1.2
Fibrinogen (mg/dl)	145	150–350
Fibrinogen/fibrin degradation products (µg/ml)	27.7	0–5
C reactive protein (mg/dl)	1.4	0.000–0.014
Lactate (mmol/l)*	2.4	0.5–1.6

As the hospital is in an SFTS endemic area, and considering her recent activities in forests and mountains, a serum nucleic acid amplification test (NAAT) for the SFTS virus was performed.

**Figure 1:**
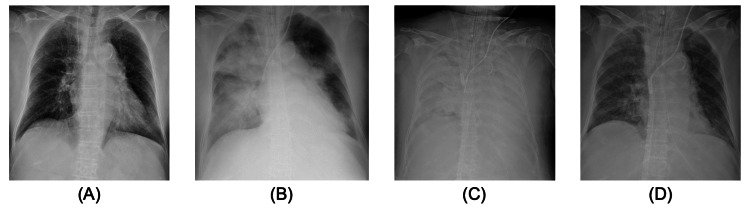
Chest X-ray findings during hospitalization Chest radiography did not reveal any abnormal findings on the day of admission (A). Ten hours after admission, chest radiography revealed bilateral diffuse alveolar opacities, suggesting acute respiratory distress syndrome (B). After starting venovenous extracorporeal membrane oxygenation, bilateral diffuse alveolar opacities deteriorated on the fourth day of hospitalization (C). On the 19th day of hospitalization, lung infiltration resolved, suggesting improvement in acute respiratory distress syndrome (D).

We administered meropenem and minocycline for suspected bacterial or rickettsial infection. We also administered 60 mg of prednisolone daily for confirmed hemophagocytic syndrome associated with the underlying cytokine storm. Ten hours after admission, her consciousness deteriorated to E3V3M5 on GCS. Her oxygenation deteriorated, requiring 8 L/min of oxygen via a non-rebreather face mask. Chest radiography revealed new bilateral diffuse alveolar opacities (Figure 1). Transthoracic echocardiography and physical examination revealed no signs of heart failure. ARDS was suspected and she was intubated and admitted to the intensive care unit (ICU). SFTS was diagnosed on the second hospital day with a positive NAAT.

We applied ventilation with low tidal volumes of 6-8 ml/kg-predicted body weight and high positive end-expiratory pressure (PEEP) of 15 cmH2O under prone positioning to treat the severe ARDS. We also administered a three-day course of 1 g of methylprednisolone daily for hemophagocytic syndrome. Despite the above initial treatment, respiratory function deteriorated further, and the partial pressure of oxygen (PaO2)/fraction of inspired oxygen (FiO2) ratio was less than 80, which persisted for more than six hours on the third hospital day. Therefore, we decided to administer VV-ECMO.

Femoro-jugular VV-ECMO was set at 1800-3000 rpm to achieve a blood flow of 3.5-4.0 L/min. The sweep gas flow rate was set to 2-3 L/min. We administered unfractionated heparin to maintain an activated partial thromboplastin time of 1.5, double the institutional control value. We applied ventilatory settings with a FiO2 of 0.21, tidal volume of 4-6 ml/kg-predicted body weight, respiratory rate of 8 breaths per minute, and PEEP of 10 cmH2O under deep sedation. Hemoglobin concentration was maintained at >10 g/dL with red cell transfusions.

On the third hospital day, *Moraxella nonliquefaciens* was isolated from one of two blood cultures obtained at hospital admission. The results of sputum and urine culture were negative. This result may reflect the contamination of blood culture because *M. nonliquefaciens* bacteremia is quite rare; it has only been reported in some case reports [6,7] and no pneumonia was observed on chest computed tomography at hospital admission. However, her deteriorating clinical course prompted us to treat her with a 14-day course of ceftriaxone for possible bacteremia rather than stop antibiotics. The result of the follow-up blood culture was negative.

On the fifth day of hospitalization, oliguria developed and acute kidney injury deteriorated to Kidney Disease: Improving Global Outcomes stage 3, prompting the administration of continuous renal replacement therapy.

The results of the serum (1,3)-β-D-glucan assay turned positive on the 11th day of hospitalization, whereas the results of (1,3)-β-D-glucan concentration at hospital admission were negative. We administered a 14-day course of antifungal agents (micafungin followed by liposomal amphotericin B) as a preemptive therapy for possible invasive fungal infections. The results of the serum galactomannan antigen assay and blood culture remained negative. No invasive fungal infections were diagnosed during her ICU stay.

On the 19th day of hospitalization, infiltration of both lungs on chest radiography significantly improved (Figure 1). She was successfully weaned off VV-ECMO. After decannulation, she remained on mechanical ventilation for persistent unconsciousness, and her PaO2/FiO2 ratio remained >300 under 8 cmH2O PEEP. On the 25th day of hospitalization, she was tracheostomized for long-term mechanical ventilation.

**Figure 2:**
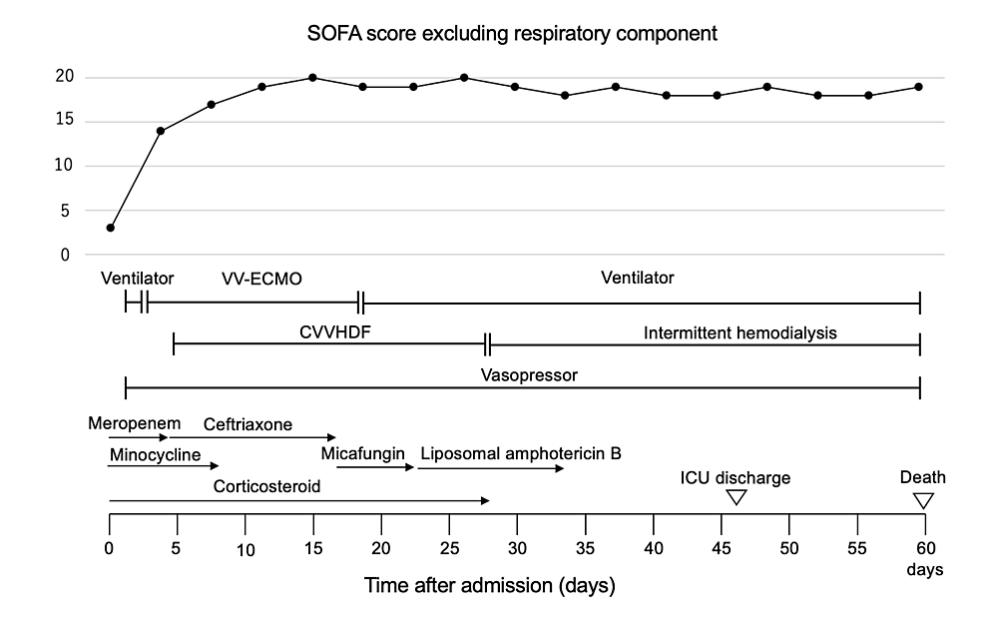
Timecourse of sequential organ failure assessment score excluding respiratory component and organ support during hospitalization SOFA, sequential organ failure assessment; VV-ECMO, venovenous extracorporeal membrane oxygenation; CVVHDF, continuous venovenous hemodiafiltration; ICU, intensive care unit.

After the amelioration of respiratory failure, other organ functions did not improve (Figure 2). Although we did not observe any diseases that could directly disturb consciousness (e.g., hepatic encephalopathy), GCS remained between E1VTM1 and E1VTM4. Hypotension requiring vasopressor administration persisted. Renal function did not improve, and the patient could not be weaned off dialysis. Thrombocytopenia persisted with platelet counts between 20,000 and 90,000/mm3. Severe liver dysfunction persisted with marked hyperbilirubinemia and coagulopathy. She died of MOF on the 60th day of hospitalization.

## Discussion

Here, we present a case of SFTS complicated by ARDS that was treated with VV-ECMO. The resolution of respiratory failure in this case suggests that ARDS due to SFTS can be improved by VV-ECMO, while other organ failures may be refractory.

SFTS is an emerging tick-borne infectious disease with a mortality rate of 11.2% to 30% [1,8]. Early, nonspecific symptoms such as fever, headache, and gastrointestinal symptoms can be followed by MOFs, and can ultimately result in death [2].

An observational study from Northeast China enrolling 115 hospitalized patients with SFTS reported the incidence of MOF, including liver (82.6%), renal (27.8%), disseminated intravascular coagulation (13%), and ARDS (6%) [3]. This study also showed that respiratory failure is associated with a higher mortality rate, despite its relatively rare incidence.

Generally, the treatment of ARDS includes respiratory management via ventilation and pharmacological management (e.g., corticosteroids). For the most severe cases of ARDS, VV-ECMO is a treatment option [9]. VV-ECMO was associated with reduced mortality in ARDS associated with H1N1 influenza and COVID-19 pneumonia [4,5]. Regarding H1N1 influenza-related ARDS, an observational study showed that the hospital mortality rate of patients referred to extracorporeal membrane oxygenation (ECMO) centers is significantly lower than that of non-ECMO referred patients after propensity score matching (24.0% vs. 46.7%) [4]. Moreover, regarding ARDS due to COVID-19 pneumonia, an international observational study showed that patients who receive ECMO have lower mortality rates than those who do not (hazard ratio = 0.55) [5]. However, to the best of our knowledge, no studies have shown the effectiveness of ECMO in patients with ARDS due to SFTS. As previous studies have shown that ARDS caused by factors other than viral pneumonia and ARDS caused by insults from extrapulmonary sites (like our case) are associated with higher mortality rates than those caused by viral pneumonia or pulmonary insults among patients treated with ECMO [10,11], it is difficult to extrapolate the abovementioned results from H1N1 influenza or COVID-19 to SFTS that has a clearly distinct pathology. H1N1 influenza and COVID-19 mainly affect the lungs as viral pneumonia, whereas SFTS often affects multiple organs. An autopsy study of a patient whose death was caused by SFTS showed SFTS virus antigens in almost all organs, including lymph nodes, liver, spleen, bone marrow, adrenals, kidneys, lungs, and heart [12]. SFTS virus directly infects most organs through viremia, which might lead to irreversible multiple organ damage in patients with SFTS. This distinct pathology of SFTS implies a different treatment effect of VV-ECMO in patients with ARDS from SFTS to that in those with influenza or COVID-19.

To our knowledge, there is only one previous case report on SFTS treated with VV-ECMO [13]. In that case, the patient was complicated with invasive pulmonary aspergillosis that required antifungal therapy. Furthermore, oxygenation deteriorated despite mechanical ventilation due to airway spasms; thus, VV-ECMO was administered. Finally, the patient died after a complicated course of invasive pulmonary aspergillosis. We could not find a case of ARDS due to SFTS treated with VV-ECMO and its role in SFTS remains unclear.

In SFTS, an increased risk of death is associated with elevated serum aspartate aminotransferase and lactate dehydrogenase levels, disseminated intravascular coagulation, seizures, altered mental status, and ARDS [2,3]. In our case, all these factors were present.

## Conclusions

We present a case of SFTS complicated by ARDS that was treated with VV-ECMO. Theoretically, VV-ECMO may contribute to the improvement of ARDS and decrease the risk of death. The resolution of respiratory failure in this case suggests that ARDS due to SFTS can be improved by VV-ECMO, while other organ failures may be refractory. However, VV-ECMO cannot, at least directly, improve the function of other organs. In fact, while respiratory function improved in our case, the patient ultimately died from MOF.

Our case underscores the importance of judicious evaluation for the indication of VV-ECMO in patients with SFTS, including assessment of the severity of not only respiratory failure but other organ failures.

## References

[1] Yu XJ, Liang MF, Zhang SY (2011). Fever with thrombocytopenia associated with a novel bunyavirus in China. N Engl J Med.

[2] Gai ZT, Zhang Y, Liang MF (2012). Clinical progress and risk factors for death in severe fever with thrombocytopenia syndrome patients. J Infect Dis.

[3] Deng B, Zhou B, Zhang S (2013). Clinical features and factors associated with severity and fatality among patients with severe fever with thrombocytopenia syndrome bunyavirus infection in Northeast China. PLoS One.

[4] Noah MA, Peek GJ, Finney SJ (2011). Referral to an extracorporeal membrane oxygenation center and mortality among patients with severe 2009 influenza A (H1N1). JAMA.

[5] Shaefi S, Brenner SK, Gupta S (2021). Extracorporeal membrane oxygenation in patients with severe respiratory failure from COVID-19. Intensive Care Med.

[6] Correa-Martínez CL, Rauwolf KK, Schuler F, Füller M, Kampmeier S, Groll AH (2019). Moraxella nonliquefaciens bloodstream infection and sepsis in a pediatric cancer patient: case report and literature review. BMC Infect Dis.

[7] Duployez C, Loïez C, Ledoux G, Armand S, Jaillette E, Wallet F (2017). A fatal endocarditis case due to an emerging bacterium: Moraxella nonliquefaciens. IDCases.

[8] Zhan J, Cheng J, Hu B (2017). Pathogens and epidemiologic feature of severe fever with thrombocytopenia syndrome in Hubei province, China. Virus Res.

[9] Combes A, Hajage D, Capellier G (2018). Extracorporeal membrane oxygenation for severe acute respiratory distress syndrome. N Engl J Med.

[10] Chiu LC, Chuang LP, Lin SW (2021). Comparisons of outcomes between patients with direct and indirect acute respiratory distress syndrome receiving extracorporeal membrane oxygenation. Membranes (Basel).

[11] Schmidt M, Bailey M, Sheldrake J (2014). Predicting survival after extracorporeal membrane oxygenation for severe acute respiratory failure. The Respiratory Extracorporeal Membrane Oxygenation Survival Prediction (RESP) score. Am J Respir Crit Care Med.

[12] Li S, Li Y, Wang Q (2018). Multiple organ involvement in severe fever with thrombocytopenia syndrome: an immunohistochemical finding in a fatal case. Virol J.

[13] Chen X, Yu Z, Qian Y, Dong D, Hao Y, Liu N, Gu Q (2018). Clinical features of fatal severe fever with thrombocytopenia syndrome that is complicated by invasive pulmonary aspergillosis. J Infect Chemother.

